# Soil microbes support Janzen’s mountain passes hypothesis: The role of local-scale climate variability along a tropical montane gradient

**DOI:** 10.3389/fmicb.2023.1135116

**Published:** 2023-03-13

**Authors:** Yifan Feng, Jianbin Wang, Jian Zhang, Xuming Qi, Wenxing Long, Yi Ding, Lan Liu

**Affiliations:** ^1^Key Laboratory of Genetics and Germplasm Innovation of Tropical Special Forest Trees and Ornamental Plants, Ministry of Education, College of Forestry, Hainan University, Haikou, China; ^2^Center for Global Change and Complex Ecosystems, Zhejiang Tiantong Forest Ecosystem National Observation and Research Station, School of Ecological and Environmental Sciences, East China Normal University, Shanghai, China; ^3^Administration Branch of Bawangling, Hainan Tropical Rain Forest National Park Service, Changjiang, China; ^4^Ecology and Nature Conservation Institute, Chinese Academy of Forestry, Beijing, China; ^5^Key Laboratory of Forest Ecology and Environment of the National Forestry and Grassland Administration, Beijing, China; ^6^Hainan Bawangling Forest Ecosystem Research Station, Changjiang, China

**Keywords:** microbial biogeography, tropical forests, climate change, cloud forests, temperature seasonality

## Abstract

Tropical montane ecosystems are the centers of biodiversity, and Janzen proposed that mountain climate variability plays a key role in sustaining this biodiversity. We test this hypothesis for soil bacteria and fungi along a 265–1,400 m elevational gradient on Hainan Island of tropical China, representing diverse vegetation types from deciduous monsoon forest to cloud forest. We found that bacterial and fungal diversity declined as elevation increased, and the dissimilarity of both groups increased with increasing separation in elevation, although changes in bacteria were larger than in fungi. Seasonal alterations and the range of soil moisture in the growing season were found to be the dominant drivers of fungal richness and Shannon diversity, whereas soil pH was the major driver of bacterial diversity. Dissimilarities of bacterial and fungal communities were best predicted by climate, particularly seasonal changes in soil temperature, with weaker influences of soil physicochemistry and vegetation. The dominant effect of seasonality in soil temperature was further detected in cloud forests, which harbored a higher proportion of unique bacterial species and dissimilarity of bacterial and fungal communities. Our findings suggest that local-climate variability plays a crucial role in structuring the distribution of soil microbial communities along a tropical montane gradient, which generally supports Janzen’s hypothesis. Such a sensitivity to climatic variability suggests that soil microbial communities along tropical montane gradients may shift in response to future climate scenarios.

## 1. Introduction

Tropical mountains cover less than 10% of the terrestrial land but support approximately half of the global biodiversity hotspots (Myers et al., 2000; Mittermeier et al., 2011; Quintero and Jetz, 2018). Various theories have been proposed to explain the extraordinarily high biodiversity in the tropics, yet a large number of species in tropical mountains remain one of the ecology’s unsolved puzzles (Mittelbach et al., 2007; Fine, 2015). Janzen (1967) proposed that mountain climates played a central role in determining the biodiversity in tropical ecosystems, which is widely known as the mountain passes hypothesis (Ghalambor et al., 2006). Focusing on seasonal variations, Janzen (1967) suggested that low climatic variability across climatic gradients creates climatic barriers for species, leading to population divergence and eventually high richness in tropical mountains. Several studies have confirmed various aspects of Janzen’s hypothesis with respect to plants and animals (Eo et al., 2008; McCain, 2009; Polato et al., 2018). However, it has remained unclear whether soil bacteria and fungi, key regulators of terrestrial biogeochemical processes, also display strong climatic variability-related patterns along different elevations in tropical montane systems.

Over the past decade, several studies have been carried out to characterize the shift in soil microbial communities along montane gradients, although tropical regions have been less examined (Hendershot et al., 2017; Looby and Martin, 2020). In these previous studies, specific groups of microbes often have unique relationships with elevation (Kivlin et al., 2017; Looby and Martin, 2020), and furthermore, community differences were best explained by elevation-related changes in soil properties, such as pH and carbon or nitrogen content, and usually not by climatic parameters *per se* (Bryant et al., 2008; Dai et al., 2021). Only a few studies along elevational transects revealed strong correlations between microbial distribution and climatic parameters (Nottingham et al., 2018a; Frindte et al., 2019; Geml et al., 2021; Ma et al., 2022). One potential explanation for the lack of a consistent climatic relationship is that most prior studies rely on low-resolution macroclimate data. For instance, mean annual temperature derived from synoptic weather stations in combination with grid-based interpolations are often used to explain diversity patterns in microbial communities, although they are known to be different from the soil climates that microbes experience (Li et al., 2013). Importantly, climatic variability such as temperature extremes within and outside the growing season can differentially affect different taxonomic groups or stages of microbial community development (Bardgett and Caruso, 2020; Ma et al., 2022). Moreover, tropical mountain climates have variability in seasonal rainfall and soil water availability (Sarmiento, 1986). These climatic features could shape microbial communities by directly mediating metabolic rates and indirectly affecting resource availability *via* controlling aboveground productivity and inputs of carbon and nitrogen to soils (Belnap et al., 2005; Körner, 2007; Keitt et al., 2016). To test Janzen’s mountain passes hypothesis for soil microbes in tropical montane systems, fine-scale climate data that reflect the climatic differences between soil cores and the variability of the climatic parameters are needed.

Tropical montane ecosystems are further characterized by frequent cloud immersion at the mid to upper elevations, which affects the climatic conditions (Bruijnzeel et al., 2011), and therefore likely regulates microbial diversity and community composition. Frequent cloud cover contributes to patterns in forest biodiversity (Hietz, 2010; Karger et al., 2021), by influencing moisture and temperature and modulating soil properties such as carbon and nitrogen content (Zimmermann et al., 2009; Fahey et al., 2016; Hernández-Vargas et al., 2019). Thus, we expect microbial communities to respond to climatic patterns. Studies on the impacts of such unique climatic phenomena on soil microbes are currently scarce, while some studies focused mainly on plant diversity patterns (Oosterhoorn and Kappelle, 2000; Fahey et al., 2016) or specific microbial groups such as ectomycorrhizal communities associated with cloud forest plants (Morris et al., 2009; Olmo-Ruiz et al., 2017).

In this study, we tested Janzen’s mountain passes hypothesis along a ~1,200 m tropical elevational gradient on Hainan Island of South China, exploring the patterns and importance of climatic variability in spatial variations of soil bacterial and fungal communities. Given the steep environmental gradient associated with increasing elevation, we expected marked shifts and directional changes in the diversity and composition of soil bacterial and fungal communities with changes in elevation (Callaway et al., 2002). We used climatic data in the soil layer from each site to calculate local-scale climatic variability, including the range of soil temperature and moisture in the growing season and the seasonality of soil temperature and moisture, among others. We expected that the local-scale soil climatic variables would play more critical roles than soil chemistry and vegetation in shaping bacterial and fungal diversity patterns and composition along the tropical elevational gradient, thereby supporting the Janzen’s hypothesis (Frindte et al., 2019; Ma et al., 2022). Likewise, we expected that soil microbial communities would respond to the unique climate in cloud forests and hold a unique pattern in diversity and composition compared with other forests in the mountain (Zimmermann et al., 2009; Velez et al., 2021).

## 2. Materials and methods

### 2.1. Study sites and soil collection

This study was conducted in the Bawangling forest region (18^o^52′-19^o^12′ N, 108^o^53′-109^o^20′ E) on Hainan Island, South China. The Bawangling Forest has a core area of 21 km^2^, and its altitude range is 200 to 1,438 m above sea level. The elevational transect consists of 12 sites at 12 different elevations from 265 to 1,400 m.a.s.l., each with a 20 × 20 m permanent sampling plot, all in old growth forest (Supplementary Table 1). These sites belong to the BEST (Biodiversity along Elevational Gradients: Shifts and Transitions) research network.^1^ Along with the increasing elevation, mean annual air temperature drops from 22.5 to 16.7°C, mean annual precipitation increases from ~1,750 mm to ~2,800 mm, and soils tend to be more acidic (pH change from 5.91 to 3.84). Precipitation is seasonal, with a rainy season (precipitation ≥100 mm per month) between May to October and a dry season (precipitation <100 mm per month) between November to April (Ding et al., 2012). The vegetation type also varies with elevation, changing from deciduous monsoon forest to lowland rain forest to montane rain forest to cloud forest. The deciduous monsoon and lowland rain forests are located at an elevation below 800 m.a.s.l. and are the most diverse forests in the Bawangling region. Woody species, including *Streblus ilicifolius*, *Terminalia hainanensis*, *Croton laevigatus*, and *Lagerstroemia balance,* while *Cyclobalanopsis patelliformis*, *Ficus altissima*, *Castanopsis tonkinensis*, *Schefflera octophylla* dominate the deciduous monsoon rainforest and lowland rain forest, respectively. The montane rain forests are from 900 to 1,100 m.a.s.l., dominated by *Dacrydium pectinatum*, *Xanthophyllum hainanense*, and *Cyclobalanopsis blakei*. Frequently enveloped by ground-level clouds and mist in combination with convective rainfall, cloud forests are located above 1,100 m.a.s.l. in the Bawangling region (Long et al., 2011). Cloud forests are dominated by woody species of *Distylium racemosum*, *Symplocos poilanei*, *Pinus fenzeliana*, *Syzygium buxifolium*, and *Engelhardia roxburghiana* (Ding et al., 2016, 2019).

Soil samples were collected from 12 sites in June 2021. At each site, four independent replicates mixed from nine evenly distributed soil cores (0–15 cm depth, 2.5 cm diameter) in a 2 m × 2 m subplot were collected. Visible roots and residues were removed. The fresh soil samples were then sieved through 2 mm mesh and subdivided into two subsamples. One was kept at 4°C to determine the physical and chemical properties, and the other was stored at −20°C until DNA extraction.

### 2.2. Soil and vegetation property measurement

Seven soil physicochemical characteristics were measured. Soil pH was measured after shaking a soil water (1, 5wt/vol) suspension for 30 min. Soil water content (%, SWC) was measured based on the sample weight before and after drying for 24 h at 105°C. Total organic carbon (TOC) and total nitrogen (TN) were determined by dichromate oxidation and titration with ferrous ammonium sulfate. Total phosphorus (TP) was determined using NaOH alkali fusion-molybdenum-antimony spectrophotometry. Soil inorganic nitrogen (IN: NH_4_^+^ and NO_3_^−^) concentrations were determined after extraction of 10 g fresh soil in 50 ml of 2 M KCl using a SmartChem 2000 discrete chemistry analyzer (WESTCO, USA).

Within a 5 m radius from the center point of each subplot, woody plants (diameter at breast height > 1 cm) were identified. Vegetation attributes included species richness (PRS), Shannon diversity (PSH), Pielou’s evenness (PEVE), and diameter at the breast height of all trees (PDBH) (Supplementary Table 2).

### 2.3. Local-scale climate data

Temperature-Moisture-Sensors (TMS, TOMST company, Czech Republic) were used to record soil temperature (0–8 cm depth) and volumetric soil moisture (0–14 cm depth) in each site (Wild et al., 2019). The TMS was able to record climate data in near-ground and soil layers continuously in 15-min intervals. In this study, we calculated the soil climatic variables through 1 year, from June 2021 to June 2022. Multiple soil climatic parameters, including mean annual soil temperature and moisture (TAM and MAM), range of soil temperature and moisture in growing season (TRanGS and MRanGS), minimum soil temperature and moisture in growing season (TMinGS and MMinGS), and seasonality of soil temperature and moisture (TSA and MSA), were calculated as previously described (Supplementary Table 2) (Ma et al., 2022).

### 2.4. Molecular analyses and sequence processing

Soil DNA was extracted within 1 week after sampling from 0.5 g wet soil samples using E.Z.N.A™ Mag-Bind Soil DNA Kit (OMEGA) following the manufacturer’s instructions. The V4–V5 hypervariable regions of bacterial 16S rRNA and ITS2 region of fungal DNA were amplified using the barcoded primer sets 515F/806R (Zhou et al., 2016) and ITS3F/ITS4R (Tedersoo et al., 2014), respectively. Each sample was amplified in triplicate. Positive PCR products were confirmed by electrophoresis. Amplicons from triplicate reactions were purified with GeneJET Gel Extraction Kit (Thermo Scientific) and mixed in equal density ratios. Sequencing was performed using Illumina Miseq (2 × 300 bp paired-end reads) platform at Majorbio company (Shanghai, China).

The QIIME2 pipeline (version 2019.10) and the DADA2 plugin with default settings were used to process raw reads (Bolyen et al., 2019). Taxonomy was assigned to representative sequences using the SILVA 132 (Quast et al., 2012) and UNITE v8.0 database (Abarenkov et al., 2010) for bacteria and fungi, respectively. Singleton ASVs were removed. All ASVs abundance tables were normalized to the smallest sample size to reduce the effect of sequence depths’ variation among samples.

### 2.5. Statistical analysis

All statistical analyses were conducted using R version 3.6.0 (R Core Team, 2019) and the R package “*vegan*” v2.4–3 (Oksanen et al., 2017) unless stated otherwise. The relationships between bacterial and fungal diversity with elevation were first examined by ordinary least squares linear regression. To determine which environmental variables most influenced microbial diversity along the elevational gradient, we used stepwise regressions with forward and backward selection using the setpAIC function in the *MASS* package (Venables and Ripley, 2013). We excluded variables collinear with other factors (variation inflation factors >10), yielding six soil, three vegetation, and five climatic variables. We calculated the relative effect of variables in the final models as the *R*^2^ contribution averaged over orderings among the important predictors using the *relaimpo* package (Grömping, 2007). The importance of individual predictors in the final model was visualized after controlling for all other predictors using the avPlots function in the *car* package (Fox and Weisberg, 2011).

Mantel tests, non-metric multidimensional scaling (NMDS), and variation partitioning analysis were used to assess the relationships between microbial compositions and environmental variables. Bray-Curtis and Jaccard dissimilarity were used to calculate the community composition. Euclidean distance was used to calculate the difference in environmental variables. After NMDS modeling determined the significant environmental factors, variation partitioning analysis was conducted using four soil variables (soil pH, SWC, TP, and NH4), two vegetation attributes (PEVE and PDBH), and five climatic parameters (MAM, TRanGS, MRanGS, TSA, and MSA).

The differences in microbial communities (diversity, composition, and relative abundance of dominant phyla) between cloud forests from elevational ranges of 1,200–1,400 m.a.s.l. and other elevational ranges of 265–502 m.a.s.l., 594–800 m.a.s.l., and 904–1,000 m.a.s.l. were compared by one-way ANOVA followed by the Dunnett’s test. Mantel tests were also used to examine the relationships between the bacterial and fungal composition with environmental variables in the cloud forests.

## 3. Results

### 3.1. Elevational patterns of soil microbial diversity and community composition

**Figure 1:**
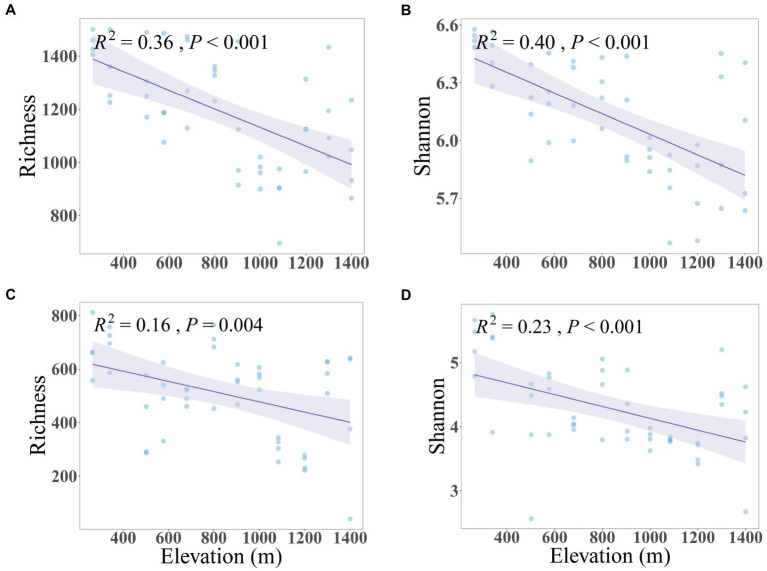
Linear relationships between soil bacterial **(A,B)** and fungal **(C,D)** diversity and elevation. The solid lines and confidence intervals show predicted relationships and 95% confidence intervals from ordinary least squares linear regression.

**Figure 2:**
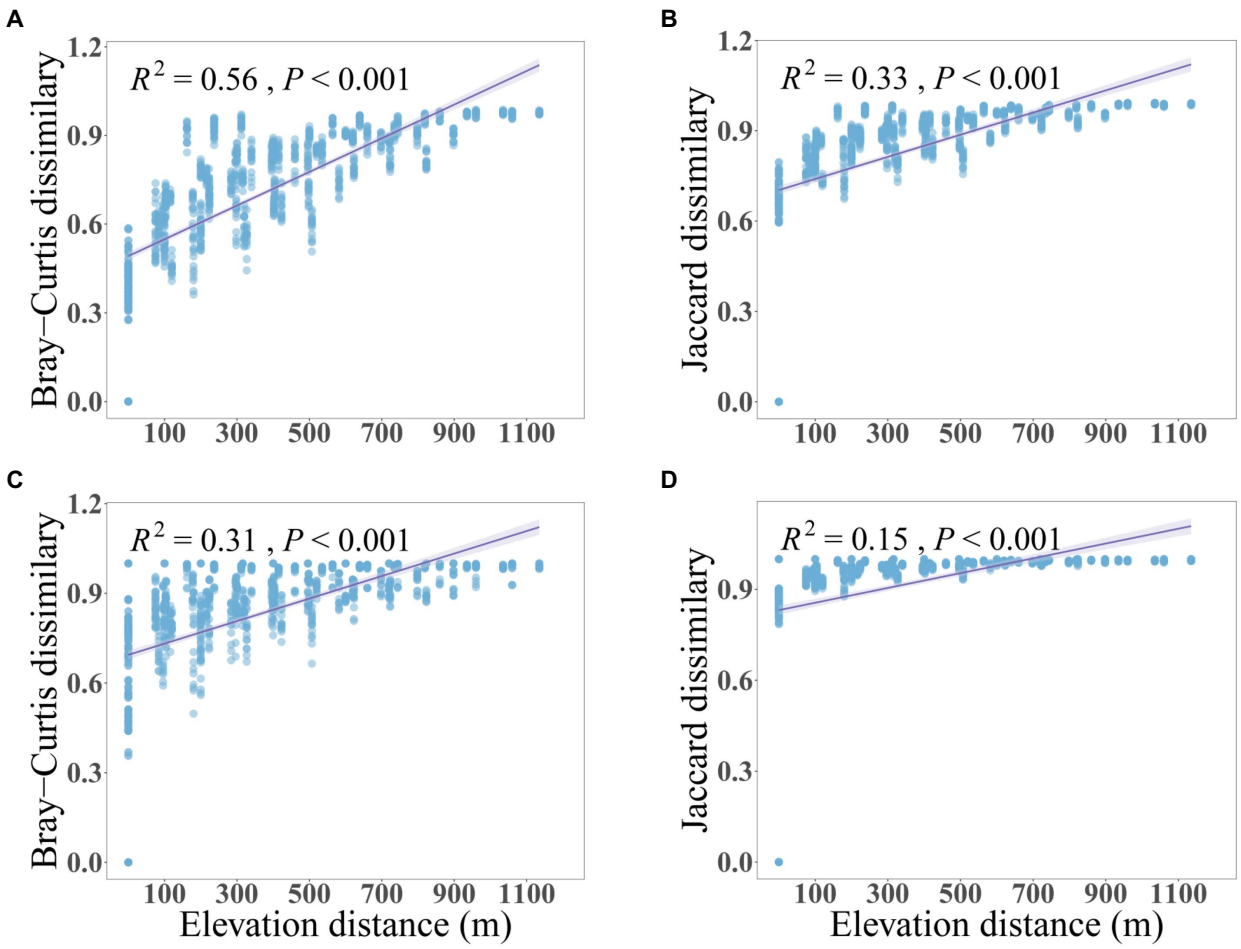
The relationships between compositional dissimilarity of soil bacteria **(A,B)** and fungi **(C,D)** with elevation differences. The solid lines represent the fitted linear regressions.

The soil microbial community along the mountain gradient in the Bawangling forest region was highly diverse, with 2,648,200 and 1,904,098 high-quality sequences assigned to 57,519 and 25,175 bacterial and fungal ASVs, respectively. Both bacterial and fungal richness and Shannon diversity declined significantly with increased elevation, with bacteria showing a stronger trend than fungi (Figure 1). Similarly, dissimilarity of bacterial and fungal communities differed significantly with elevation (PERMANOVA of Bray–Curtis dissimilarity: *F* = 11.76 and 3.83, *R*^2^ = 0.78 and 0.54, *p* = 0.001 and 0.001, respectively. PERMANOVA of Jaccard dissimilarity: *F* = 6.07 and 2.55, *R*^2^ = 0.65 and 0.44, *p* = 0.001 and 0.001, respectively). The Bray-Curtis and Jaccard dissimilarities were positively correlated to elevation and were characterized by linear relationships whereby the community dissimilarity tended toward a maximum (dissimilarity = 1) with increased elevational separation (Figure 2). The elevation-related changes were also observed in the dominant phyla. For bacteria, Verrucomicrobiota (7.27%) and Chloroflexi (4.75%) decreased linearly and Acidobacteria (16.05%) increased linearly as elevation increased. For fungi, Ascomycota (51.55%) and Mortierellomycota (4.98%) displayed significantly reduced and increased relative abundance with elevation, respectively (Supplementary Figure 1, Supplementary Table 3).

**Figure 3:**
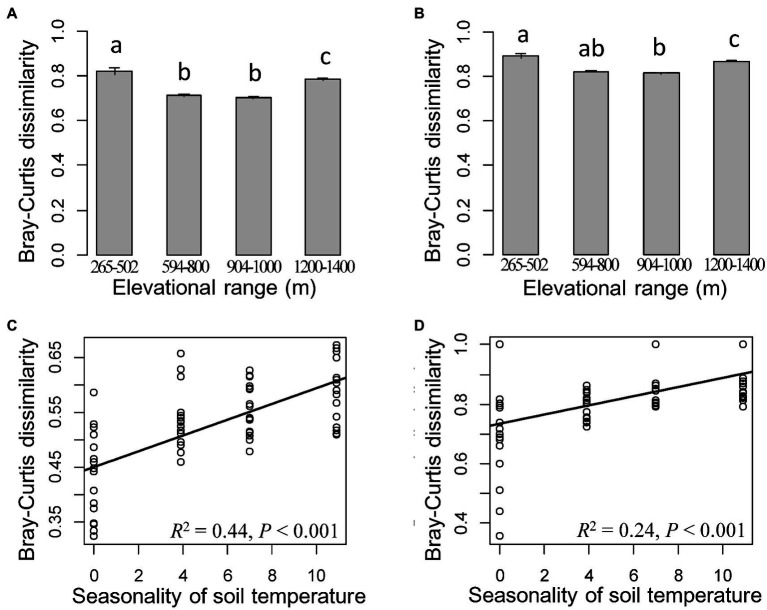
Compositional dissimilarity among cloud forests from elevational range of 1,200–1,400 m and other elevational ranges for bacteria **(A)** and fungi **(B)**. Differences are significant when no same letter exists between elevational ranges (one-way ANOVA followed by Dunnett’s test; *p* < 0.05). The most important predictor of compositional dissimilarity for bacteria **(C)** and fungi **(D)** in cloud forests from elevational range of 1,200–1,400  m.a.s.l. The solid lines represent the fitted linear regressions.

**Figure 4:**
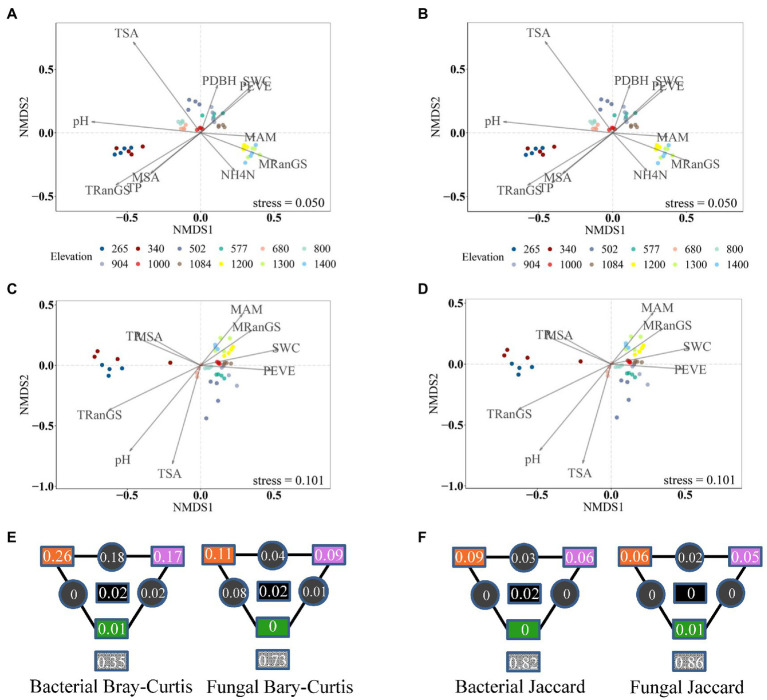
Non-metric multidimensional scaling (NMDS) of compositional dissimilarity for bacteria **(A,B)** and **(C,D)** fungi using Bray-Curtis **(A,C)** and Jaccard **(B,D)** dissimilarity. Colors show the elevation of the soil sample. The strength of statistically significant (*p* < 0.05) explanatory variables is shown with solid arrows. **(E,F)** Partitioning of bacterial and fungal variance in compositional dissimilarity among important soil climatic factors (orange), soil chemistry (purple), vegetation (green), and their interactions (black). The values in gray squares are unexplained variances.

Bacterial and fungal communities in cloud forests from elevational ranges of 1,200–1,400 m.a.s.l. harbored a higher percentage of unique ASVs compared to the other three elevational ranges (Supplementary Figure 2; Supplementary Table 4). High bacterial diversity was also detected in those forests (Supplementary Table 4). Meanwhile, bacterial and fungal communities from cloud forests were clearly separated from other forests and displayed a significantly different within-group variation compared to the other three elevational ranges (Figures 3A,B, 4A–D).

### 3.2. The environmental drivers of microbial diversity and community composition

**Figure 5:**
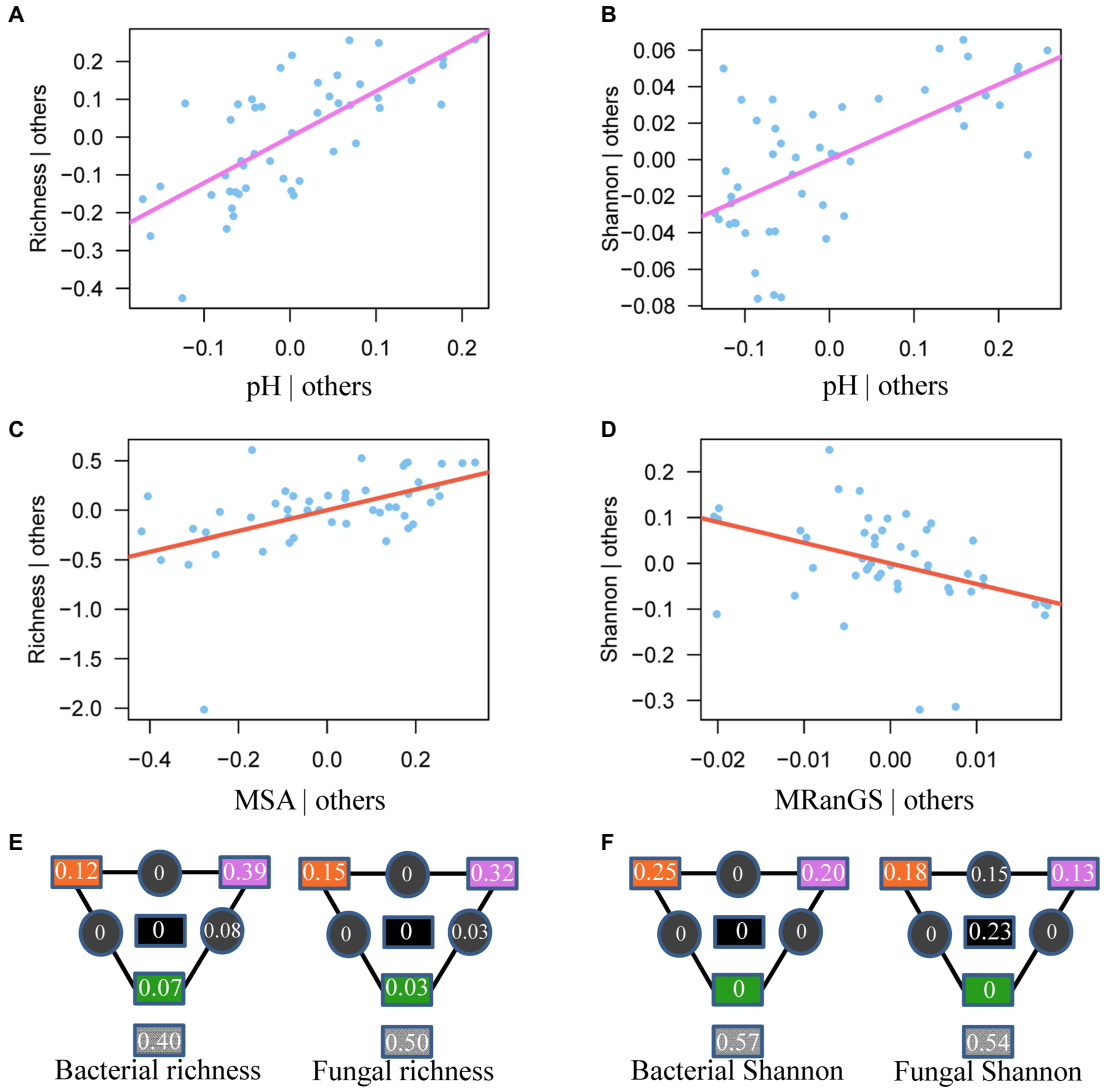
The most important predictor of bacterial **(A,B)** and fungal **(C,D)** diversity. Solid lines show the relationships between the best predictor based on AIC selection and microbial diversity after controlling for the effects of other significant variables in the model in Supplementary Table 5. MSA: Seasonality of soil moisture; MRanGS: Range of soil moisture in growing season. **(E,F)** Partitioning of bacterial and fungal diversity variance among important climatic factors (orange), soil chemistry (purple), vegetation (green), and their interactions (black). The values in gray squares are unexplained variances.

Compared to soil and vegetation properties, climatic variables had the greatest, significant influence on the fungal diversity and both bacterial and fungal community composition, whereas bacterial richness and Shannon diversity were mainly influenced by soil properties, particularly pH (*R*^2^ = 0.43 and 0.35 after the effect of other significant predictor variables were controlled; Figures 4, 5 and Supplementary Table 5). Fungal richness and Shannon diversity were most strongly influenced by MSA and MRanGS (*R*^2^ = 0.18 and 0.21, *p* < 0.01), respectively. The community composition of bacteria and fungi, measured as Bray-Curtis and Jaccard dissimilarity, was best predicted by TSA (Figures 4A–D). The importance of these environmental attributes was verified by Pearson’s correlation and Mantel test (*p* < 0.05, Supplementary Table 6). In addition, TSA was identified as the dominant factor that explained the dissimilarity of bacterial and fungal communities in the cloud forests (*R*^2^ = 0.44 and 0.24, *p* < 0.001; Figures 3C,D; Supplementary Table 7).

Among climatic, edaphic, and vegetation factors, the influence of selection by climatic factors on fungal diversity and bacterial and fungal composition was strongest (Figures 4E,F, 5E,F). Climatic effects were also observed at the phylum level (Supplementary Table 3). For instance, bacterial phyla of Actinobacteria, Acidobacteria, and Verrucomicrobiota were most strongly correlated with TSA, MAM, and MRanGS, respectively. Further, soil physicochemical attributes were the main factor in the selection process that influenced bacterial richness and Shannon diversity, as well as dominant bacterial phyla of Proteobacteria and Planctomycetota and fungal phyla of Ascomycota and Mortierellomycota. Vegetation attributes of PEVE and PDBH uniquely explained 0.04–0.06 compositional variations in bacterial and fungal communities and 0.03–0.07 variations in bacterial diversity. They also influenced the bacterial phyla of Proteobacteria and fungal phyla of Ascomycota and Basidiomycota (Supplementary Table 3).

## 4. Discussion

Our first expectation that the microbial diversity and composition display directional changes along the tropical montane gradient was verified. We revealed that both bacterial and fungal diversity, measured as richness and Shannon index, declined as elevation increased, and the dissimilarity of bacterial and fungal communities increased with increased elevation differences (Figures 1, 2). While geographical patterns of soil microbial communities have been a hot topic in recent years, few studies have examined their distribution along elevational gradients in the tropics (Wang et al., 2022). Our results demonstrated a significant role for the abiotic environment in shaping the diversity and composition of soil bacteria and fungi along the elevational gradient. Fundamentally, climatic factors, including soil temperature and moisture and certain edaphic factors, particularly pH, were strongly associated in our data with variations in bacterial and fungal diversity and composition along the montane gradient.

Our results generally supported the Janzen’s hypothesis. We confirmed that climatic variability had a strong influence on the spatial variation of soil bacterial and fungal composition and fungal diversity along a tropical montane elevational gradient. Although effects of climatic variability on microbial diversity and composition have been observed in ecosystems such as forests and grasslands (Rasche et al., 2011; Shi et al., 2020), previous studies have often found soil properties such as pH and carbon or nitrogen content as the dominant factors that explained microbial distribution along mountainsides (Singh et al., 2012; Shen et al., 2013; Zhang et al., 2015). Using local-scale soil temperature and moisture data instead of macroclimate data might be the key to resolving this contradiction. Meanwhile, climatic differences between soil cores reflect potential climate extremes that determine microbial survival and climatic variability that meditate compositional shifts in microbial communities directly and indirectly (Bell et al., 2009; Keitt et al., 2016). Specifically, the range of soil moisture in the growing season and moisture seasonality were the main drivers of fungal diversity along our elevational gradient. There is evidence indicating that fungi, particularly fungal spores, are more sensitive than bacteria to changes in soil moisture (Griffin, 1963; Kaisermann et al., 2015), and in some cases fungal abundance based on qPCR of 18S rRNA gene was more reduced by drought (Cregger et al., 2012). Because the daily or circadian climate cycle is markedly higher than the differences in climate from month to month in tropical regions (Janzen, 1967; Sarmiento, 1986), the negative correlation between fungal diversity and the range of moisture during the growing season suggests that dry soil conditions negatively influence fungal diversity (He et al., 2017; Preece et al., 2019), whereas the positive relationship with seasonal variation in soil moisture suggests niche partitioning with respect to soil moisture could be facilitating fungal diversity (Kaisermann et al., 2015; Větrovský et al., 2019). In addition, soil temperature directly affects microbial metabolism and the seasonal changes are closely linked to short- and medium-term variations in the quantity and quality of resources entering the soil (Krave et al., 2002; Cookson et al., 2006; Bell et al., 2009), which further correlated with compositional variation of both bacterial and fungal communities.

Besides climatic attributes, soil properties as pH, NH_4_^+^, TP, water content, and plant evenness and tree biomass storage were also crucial for shaping microbial diversity and community composition. Consistently, soil pH has been documented as the key driver of bacterial communities along elevational gradients globally (Wang et al., 2022). It has also been documented that increased nitrogen limitation could lead to more fungi-dominated microbial communities at higher elevations (Fierer et al., 2009; Nottingham et al., 2018b). Generally, microbial composition and abundance depend on soil nutrient availability (Lozupone and Knight, 2007; Jesus et al., 2009). Meanwhile, plant attributes of diversity and biomass (simplified as diameter at breast height of trees) may regulate bacterial and fungal communities by determining the quantity and quality of the litter and root exudate supply (such as C or N source) and by modifying the soil physical environment (Wallenstein et al., 2007). Particularly, a more even plant community should result in a more even distribution of roots. There is evidence that plants can alter root growth strategies in response to variations in their neighbors and soil nutrients (Wambsganss et al., 2021). An even distribution of roots, thus, should maximize the volume of the soil explored by plant roots and maximize the number of microhabitats available to the soil microbial community.

Biodiversity in cloud forests is still largely under-documented and poorly understood (Karger et al., 2021), particularly soil microbial communities. Our data revealed a more diverse bacterial community and divergent composition of both bacterial and fungal communities in the cloud forests (Figure 3A,B, 4A–D; Supplementary Figure 2). The findings are consistent with our expectations and suggest that the microbial community, especially bacteria in the cloud forests, was substantially different from those of other forests along the montane gradient. Notably, humid cloud forest soils with less fluctuation in temperature (decreased seasonality of soil temperature and increased mean annual soil moisture in Supplementary Table 2) may foster unique microbial communities. On the one hand, the relatively stable environments, compared to other elevational ranges, may enable increased survival and even speciation in microbial taxa, especially in bacterial communities (Pellissier, 2015), which deserve further investigation. On the other hand, microbial life-history strategies may also explain part of the phenomenon. For instance, phyla of Acidobacteria prefer soil with high moisture, whereas Chloroflexi prefer warmer soil conditions (Jones et al., 2009; Oliverio et al., 2017; Pérez Castro et al., 2019). Hence, Acidobacteria may be particularly favored in the cloud forest soils, but Chloroflexi was reduced (Supplementary Table 4).

As tropical montane ecosystems may face considerable threats from future climate change (Pounds et al., 1999; Foster, 2001), disentangling the role of climate in the distribution of biotic communities along tropical montane gradients is highly valuable for predicting the consequences of climate change. Here, we show that soil bacterial and fungal communities on tropical montane displayed monotonic changes with increasing elevation and that bacterial and fungal diversity and community composition were largely predicted by climatic variability. Since global warming is expected to increase seasonal variability and alter daily temperature extremes (Karl et al., 1991; Wang and Dillon, 2014), climate change is likely to shift microbial communities in tropical montane systems and impact terrestrial biogeochemical cycling, particularly in cloud forests.

Finally, we acknowledge that the results reported here come from only one gradient study. Tropical montane systems are highly variable in climatic conditions due to differences in topography, cloud climatology, and geographic location (Janzen, 1967; Sarmiento, 1986). This climatic variability could lead to divergent patterns in soil microbial diversity and composition but is also a crucial ecological characteristic for life and survival of microorganisms. As such, similar studies in other tropical elevational gradients are needed for a more unified understanding of mountain microbes. Our work shows how the climatic variability perspective can provide new insights into microbial ecological studies.

## Data availability statement

The data presented in the study are deposited in the National Center for Biotechnology Information (NCBI) Sequence Read Archive (SRA) repository, accession number PRJNA903527.

## Author contributions

LL, YD, and WL contributed to the conception and design of the study. YF, JW, XQ, and LL participated in the sample collection and processing. YF, JZ, YD, and LL contributed to the data analysis, methodology, visualization, and validation. YF wrote the original draft. LL organized the field expedition and acquired the funding. All authors contributed to the review, editing, and approval of the final submitted version.

## Funding

This research was financially supported by Hainan Natural Science Fund (322RC581 to LL), Hainan University (KYQD(ZR)21116 to LL), and Fundamental Research Funds for the Central Non-profit Research Institution of Chinese Academy of Forestry (CAFYBB2022SY024 to YD).

## Conflict of interest

The authors declare that the research was conducted in the absence of any commercial or financial relationships that could be construed as a potential conflict of interest.

## Publisher’s note

All claims expressed in this article are solely those of the authors and do not necessarily represent those of their affiliated organizations, or those of the publisher, the editors and the reviewers. Any product that may be evaluated in this article, or claim that may be made by its manufacturer, is not guaranteed or endorsed by the publisher.

## Footnotes

^1^ https://BEST-mountains.org
