# Supplementary material for: Soil microbes support Janzen’s mountain passes hypothesis: The role of local-scale climate variability along a tropical montane gradient
Source: Front Microbiol. 2023 Mar 13;14:1135116. doi: 10.3389/fmicb.2023.1135116 (PMC10040759; doi:10.3389/fmicb.2023.1135116)
Supplement: Supplementary file 1 [file Data_Sheet_1.docx]

Supplementary Material

**Soil microbes support Janzen's mountain passes hypothesis: the role of local-scale climate variability along a tropical montane gradient**

**Yifan Feng1, Jianbin Wang1, Jian Zhang2, Xuming Qi3, Wenxing Long1,4*, Yi Ding 4,5* and Lan Liu1***

*** Correspondence:**

Lan Liu*, liulan_sh@qq.com

Yi Ding*, dingyi@caf.ac.cn

Wenxing Long*, oklong@hainanu.edu.cn

# Supplementary Figures and Tables

Supplementary Table 1 Elevation and location of sampling sites along a tropical elevational gradient on Hainan Island, South China.

| **Elevation (m.a.s.l)** | **Position** |
| --- | --- |
| 265 | 109°05′39.2″E, 19°06′17.4″N |
| 340 | 109°06′32.9″E, 19°06′38.1″N |
| 502 | 109°07′11.9″E, 19°06′58.3″N |
| 577 | 109°07′13.4″E, 19°06′52.7″N |
| 680 | 109°08′20″E, 19°06′19″N |
| 800 | 109°07′45.5″E, 19°04′15.6″N |
| 904 | 109°12′53.5″E, 19°06′44.5″N |
| 1000 | 109°11′21.5″E, 19°05′39.8″N |
| 1084 | 109°12′23.4″E, 19°04′41.1″N |
| 1200 | 109°12′56.2″E, 19°05′24.5″N |
| 1300 | 109°12′43.7"E, 19°04′58.9"N |
| 1400 | 109°12′40.8"E, 19°05′12.2"N |

Supplementary Table 2 Summary of used environmental parameters and their Pearson correlations (r) with elevation. Significance levels are: **P* < 0.05, ** *P* < 0.01, and *** *P* < 0.001.

| **Category** | **Environmental variables** | **Abbreviation** | **Mean ± SD** | **r** | **Units** |
| --- | --- | --- | --- | --- | --- |
| **Climate** | Mean annual soil temperature | TAM | 19.57±1.85 | -0.98*** | ^o^C |
|  | Mean annual soil moisture | MAM | 0.27±0.12 | 0.53** | % |
|  | Range of soil temperature in growing season | TRanGS | 0.81±0.40 | -0.63*** | ^o^C |
|  | Range of soil moisture in growing season | MRanGS | 0.04±0.01 | 0.54*** | % |
|  | Minimum soil temperature in growing season | TMinGS | 21.03±1.62 | -0.94*** | ^o^C |
|  | Minimum soil moisture in growing season | MMinGS | 0.16±0.11 | 0.47** | % |
|  | Seasonality of soil temperature | TSA | 228±13.07 | -0.84*** | ^o^C |
|  | Seasonality of soil moisture | MSA | 4.34±1.50 | -0.24 | % |
| **Soil** | Soil pH | pH | 4.53±0.68 | -0.86******* | \ |
|  | Soil water content | SWC | 17.21±8.11 | 0.51******* | % |
|  | Total organic C | TOC | 2.72±1.81 | 0.44****** | g/kg |
|  | Total N | TN | 0.18±0.08 | 0.26 | g/kg |
|  | Total P | TP | 0.15±0.10 | -0.25 | g/kg |
|  | NH_4_^+^ | NH_4_ | 15.63±14.48 | 0.38****** | mg/kg |
|  | NO_3_^-^ | NO_3_ | 16.06±10.56 | 0.25 | mg/kg |
| **Plant** | Species richness | PRS | 25.04±9.74 | 0.15 | \ |
|  | Shannon-Wiener index | PSH | 2.66±0.54 | 0.32 | \ |
|  | Pielou's evenness | PEVE | 0.64±0.23 | 0.27 | \ |
|  | Diameter at breast height | PDBH | 412.84±342.55 | 0.07 | cm |

Supplementary **Table 3** Pearson correlations between dominant bacterial and fungal phyla and environmental variables. Significance levels are: **P* < 0.05, ** *P* < 0.01, and *** *P* < 0.001.

| **Phylum** | Elevation | TAM | MAM | TRanGS | MRanGS | TMinGS | MMinGS | TSA | MSA |
| --- | --- | --- | --- | --- | --- | --- | --- | --- | --- |
| Proteobacteria | 0.22 | -0.22 | -0.06 | -0.36* | 0.24 | -0.15 | 0.07 | -0.09 | -0.14 |
| Actinobacteria | 0.22 | -0.20 | -0.14 | -0.07 | 0.15 | -0.21 | -0.17 | -0.46** | -0.21 |
| Acidobacteria | 0.42** | -0.42** | 0.54*** | -0.18 | 0.20 | -0.41** | 0.50*** | -0.21 | 0.01 |
| Planctomycetota | 0.20 | -0.19 | 0.14 | -0.10 | 0.10 | -0.15 | -0.04 | -0.01 | -0.12 |
| Verrucomicrobiota | -0.48*** | 0.45** | 0.12 | 0.34* | -0.58*** | 0.38* | 0.22 | 0.48*** | 0.42** |
| Chloroflexi | -0.75*** | 0.73*** | -0.25 | 0.26 | -0.40** | 0.77*** | -0.15 | 0.74*** | 0.04 |
| Ascomycota | -0.35** | 0.36* | -0.22 | 0.42** | -0.36* | 0.27 | -0.18 | 0.13 | 0.11 |
| Basidiomycota | 0.07 | -0.08 | 0.11 | -0.16 | 0.19 | -0.06 | -0.04 | 0.09 | 0.11 |
| Mucoromycota | 0.28 | -0.27 | 0.09 | -0.34* | 0.15 | -0.15 | 0.31 | -0.13 | -0.31* |
| Mortierellomycota | 0.42** | -0.44** | 0.24 | -0.42** | 0.28 | -0.38* | 0.33* | -0.37* | -0.22 |
| Rozellomycota | 0.16 | -0.06 | -0.07 | 0.08 | 0.05 | -0.14 | 0.07 | -0.18 | -0.13 |

| **Phylum** | **pH** | **TOC** | **TN** | **TP** | **NH_4_**^+^ | **NO_3_**^-^ | **PSR** | **PSH** | **PEVE** | **PDBH** |
| --- | --- | --- | --- | --- | --- | --- | --- | --- | --- | --- |
| Proteobacteria | -0.33* | -0.05 | -0.17 | **-0.42*** | -0.02 | 0.02 | 0.19 | 0.35* | 0.32* | -0.11 |
| Actinobacteria | 0.01 | 0.18 | 0.16 | 0.13 | 0.07 | -0.04 | 0.06 | -0.13 | -0.16 | 0.27 |
| Acidobacteria | -0.48*** | 0.26 | 0.30* | 0.18 | 0.17 | 0.39** | 0.02 | 0.23 | 0.16 | -0.13 |
| Planctomycetota | -0.05 | 0.05 | -0.15 | -0.36* | 0.28 | -0.40** | -0.33* | -0.08 | 0.24 | -0.18 |
| Verrucomicrobiota | 0.21 | -0.23 | 0 | -0.35* | -0.27 | 0.29** | -0.03 | -0.19 | -0.27 | -0.02 |
| Chloroflexi | 0.56*** | -0.36* | -0.20 | 0.16 | -0.29 | -0.15 | -0.20 | -0.29 | -0.20 | 0.14 |
| Ascomycota | 0.45** | -0.06 | -0.05 | 0.11 | 0 | -0.18 | -0.01 | -0.35* | -0.38* | 0.39** |
| Basidiomycota | -0.16 | -0.09 | -0.09 | -0.08 | -0.03 | 0.03 | -0.22 | 0.07 | 0.19 | -0.44** |
| Mucoromycota | -0.36* | 0.25 | 0.2 | -0.21 | 0.07 | 0.15 | 0.18 | 0.39** | 0.29 | -0.16 |
| Mortierellomycota | -0.47*** | 0.15 | 0.21 | 0.16 | 0 | 0.34** | 0.31* | 0.39** | 0.29 | -0.07 |
| Rozellomycota | -0.19 | 0.1 | 0.17 | 0.27 | 0.05 | 0.13 | -0.05 | -0.10 | -0.02 | 0.02 |

Supplementary Table 4 Welch’s t-tests comparing diversity, composition, and the mean relative abundances of dominant phyla across elevational soils. Cloud forests are located in the elevational rages of 1,200-1,400 m. Differences are significant when no same letter exists between elevational ranges (one-way ANOVA followed by Dunnett's test; *P* < 0.05).

| **Group** | **Category** | **265-502 m** | **594-800 m** | **904-1000 m** | **1,200-1,400 m** |
| --- | --- | --- | --- | --- | --- |
| Bacteria | Richness | 1358±358a | 1292±292a | 982±177.1b | 1111±163.1bc |
|  | Shannon | 6.37±.37a | 6.24±.24a | 5.93±0.23b | 5.93±0.33bc |
|  | Bray-Curtis | 0.82±0.82a | 0.72±0.72b | 0.70±0.70b | 0.79±0.70c |
|  | Jaccard | 0.89±.89a | 0.82±.82b | 0.81±.81b | 0.87±.87c |
| Phylum | Proteobacteria | 25.74±8.29a | 35.96±6.76b | 33.13±6.34bc | 30.27±3.05ac |
|  | Actinobacteria | 22.35±6.87a | 14.55±3.06b | 18.54±5.48a | 27.21±8.74a |
|  | Acidobacteria | 13.58±3.34a | 13.98±2.55a | 19.53±3.89b | 17.11±3.82b |
|  | Planctomycetota | 8.77±4.87ab | 11.2±4.51ab | 8.09±3.86a | 12±4.73ba |
|  | Verrucomicrobiota | 8.37±2.56a | 9.71±4.81a | 7.57±2.39ab | 3.43±1.48c |
|  | Chloroflexi | 7.53±2.15a | 5.44±1.52b | 5.03±2.47b | 0.98±0.37c |
| Fungi | Richness | 589.6±169.8 | 549.8±124.2 | 475.2±131.4 | 420.2±209.2 |
|  | Shannon | 4.76±0.94a | 4.38±0.45ab | 3.95±0.34b | 4.02±0.68b |
|  | Bray-Curtis | 0.89±.89a | 0.82±.821ab | 0.81±.811b | 0.87±.871c |
|  | Jaccard | 0.97±.97a | 0.92±.92dab | 0.90±.90b | 0.94±.94c |
| Phylum | Ascomycota | 62.72±17.9a | 50.71±13.45ab | 41.26±8.11b | 51.53±14.47ab |
|  | Basidiomycota | 31.07±19.6 | 42.71±14.27 | 38.07±10.73 | 33.1±17.13 |
|  | Mucoromycota | 1.07±1.12a | 3.52±4.25a | 10.37±7.40b | 4.97±8.16ab |
|  | Mortierellomycota | 0.26±0.25a | 0.62±0.58a | 7.45±4.97b | 4.82±7.56ab |
|  | Unclassified_fungi | 3.09±1.63 | 1.37±0.74 | 1.47±1.04 | 4.01±5.82 |
|  | Rozellomycota | 0.82±0.62 | 0.46±0.31 | 1.06±0.62 | 1.07±0.68 |

Supplementary Table 5 Environmental variables predicting diversity of bacteri and fungi in cloud forests from elevational ranges of 1,200-1,400 m. Table shows the proportion of variance explained (R^2^), sign of the relationship (+ / -), and significance codes (* ≤ 0.05, ** ≤ 0.01, *** ≤ 0.001) for multivariate regression models.

| **Variable** | **Bacteria** | | **Fungi** | |
| --- | --- | --- | --- | --- |
|  | Richness | Shannon | Richness | Shannon |
| TAM | - | - | - | - |
| MAM | 0.07 (+)* | ns | ns | ns |
| TRanGS | ns | ns | 0.07 (-)** | ns |
| MRanGS | ns | ns | ns | 0.21 (-)** |
| TMinGS | - | - | - | - |
| MMinGS | - | - | - | - |
| TSA | ns | ns | 0.08 (+)*** | ns |
| MSA | 0.06 (+)** | 0.16 (+)*** | 0.18 (+)*** | 0.05 (+)* |
| pH | 0.43 (+)*** | 0.35 (+)*** | ns | ns |
| SWC | ns | ns | 0.06(-)** | 0.17 (-)** |
| TOC | - | - | - | - |
| TN | ns | ns | ns | ns |
| TP | ns | ns | 0.13 (+)* | ns |
| NH_4_^+^ | ns | ns | 0.04 (+)* | 0.03 (+)** |
| NO_3_^-^ | ns | ns | ns | ns |
| PSR | 0.08 (-)* | 0.02 (-)* | ns | ns |
| PSH | - | - | - | - |
| PEVE | ns | ns | ns | ns |
| PDBH | ns | ns | ns | ns |

“-“: Variables excluded from multivariate regression models due to collinearity.

Supplementary Table 6 Relationships between environmental variables and microbial diversity and compositional dissimilarity using Pearson correlation and Mantel test. Significance levels are: **P* < 0.05, ** *P* < 0.01, and *** *P* < 0.001.

| **Variables** | **Bacteria** | | **Fungi** | | **Bacteria** | | **Fungi** | |
| --- | --- | --- | --- | --- | --- | --- | --- | --- |
|  | **Richness** | **Shannon** | **Richness** | **Shannon** | **Bray-**  **curtis** | **Jaccard** | **Bray-**  **curtis** | **Jaccard** |
| Elevation | -0.60** | -0.63*** | -0.42** | -0.48** | 0.76*** | 0.73*** | 0.60*** | 0.66*** |
| TAM | 0.57*** | 0.64*** | 0.40** | 0.48*** | 0.76*** | 0.73*** | 0.60*** | 0.66*** |
| MAM | -0.18 | -0.22 | 0.07 | -0.13 | 0.15** | 0.16** | 0.16** | 0.25*** |
| TRanGS | 0.59*** | 0.59*** | 0.39** | 0.47*** | 0.37*** | 0.37*** | 0.40*** | 0.35*** |
| MRanGS | -0.35* | -0.46*** | -0.52*** | -0.56*** | 0.26*** | 0.30*** | 0.21** | 0.32*** |
| TMinGS | 0.46** | 0.55** | 0.33 | 0.41** | 0.63*** | 0.60*** | 0.50*** | 0.55*** |
| MMinGS | -0.37* | -0.32* | -0.03 | -0.17 | 0.17** | 0.19** | 0.19** | 0.31*** |
| TSA | 0.55*** | 0.53*** | 0.33* | 0.34* | 0.55*** | 0.52*** | 0.41*** | 0.50*** |
| MSA | 0.31* | 0.37* | 0.43** | 0.33* | 0.19** | 0.25** | 0.21** | 0.30*** |
| pH | 0.65*** | 0.58*** | 0.31* | 0.46** | 0.50*** | 0.48*** | 0.50*** | 0.46*** |
| TOC | -0.59*** | -0.51*** | -0.25 | -0.41** | 0.09 | 0.11 | -0.01 | 0.07 |
| TN | -0.32* | -0.13 | 0.02 | -0.13 | 0.06 | 0.08 | -0.03 | 0.09* |
| TP | -0.33* | -0.09 | 0.15 | -0.07 | 0.24** | 0.23*** | 0.10 | 0.27*** |
| NH_4_^+^ | -0.03 | 0.19 | 0.41** | 0.18 | 0.10 | 0.12 | 0.04 | 0.07 |
| NO_3_^-^ | -0.11 | 0 | 0.08 | -0.03 | 0.02 | 0.04 | 0.00 | 0.15** |
| PSR | -0.48*** | -0.25 | 0.05 | -0.20 | 0.07 | 0.08 | 0.03 | 0.08 |
| PSH | -0.39** | -0.25 | -0.28 | -0.18 | 0.19** | 0.20*** | 0.12 | 0.15** |
| PEVE | -0.41** | -0.33 | -0.38** | -0.31* | 0.16** | 0.17** | 0.12* | 0.15** |
| PDBH | -0.18 | -0.25 | -031* | -0.25 | 0.16* | 0.16* | 0.23* | 0.21*** |

Supplementary Table 7 Mantel test for the correlations between environmental variables and compositional dissimilarity of bacteria and fungi in cloud forests from elevational ranges of 1200 -1400 m. Significance levels are: **P* < 0.05, ** *P* < 0.01, and *** *P* < 0.001.

| **Variables** | **Bacteria** | | **Fungi** | |
| --- | --- | --- | --- | --- |
|  | **Bary-Curtis** | **Jaccard** | **Bary-Curtis** | **Jaccard** |
| TAM | **0.55***** | **0.67***** | **0.44***** | **0.50***** |
| MAM | 0.56******* | 0.72*** | 0.49******* | 0.58*** |
| TRanGS | 0.66*** | 0.82*** | 0.51*** | 0.62*** |
| MRanGS | **0.35*** | 0.36***** | 0.19 | 0.18 |
| TMinGS | **0.45*** | 0.51** | 0.19 | 0.26* |
| MMinGS | **0.48**** | 0.54** | 0.21 | 0.27* |
| TSA | 0.67** | **0.83***** | 0.50*** | **0.61***** |
| MSA | 0.46** | **0.50**** | 0.23***** | **0.25** |
| pH | 0.46** | 0.40** | 0.10 | **0.10** |
| TOC | 0.47** | 0.52****** | 0.18 | 0.26 |
| TN | 0.48** | 0.57****** | 0.21 | 0.30 |
| TP | 0.43** | 0.48** | 0.19 | 0.27 |
| NH_4_^+^ | 0.43** | 0.40** | 0.12 | 0.18 |
| NO_3_^-^ | 0.42** | 0.58** | 0.36 | 0.43 |
| PSR | -0.04 | -0.01 | 0.33 | 0.40* |
| PSH | 0.04 | 0.01 | 0.02 | 0.01 |
| PEVE | 0.20 | 0.29* | 0.24 | 0.24 |
| PDBH | -0.10 | -0.04 | 0.44 | 0.54* |


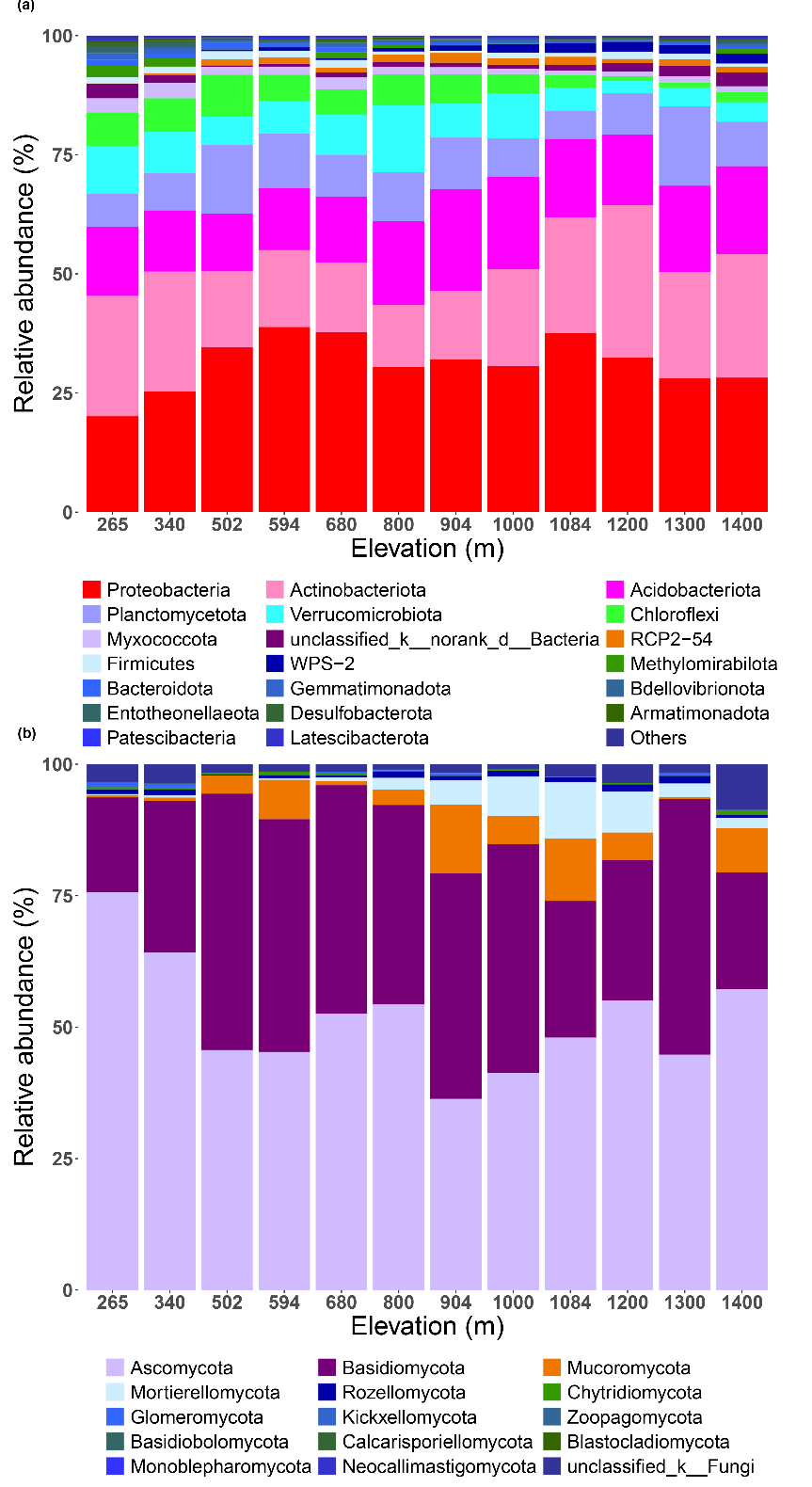


**Supplementary Figure 1.** Relative abundance of the dominant soil bacterial (a) and fungal (b) phyla at different elevations.


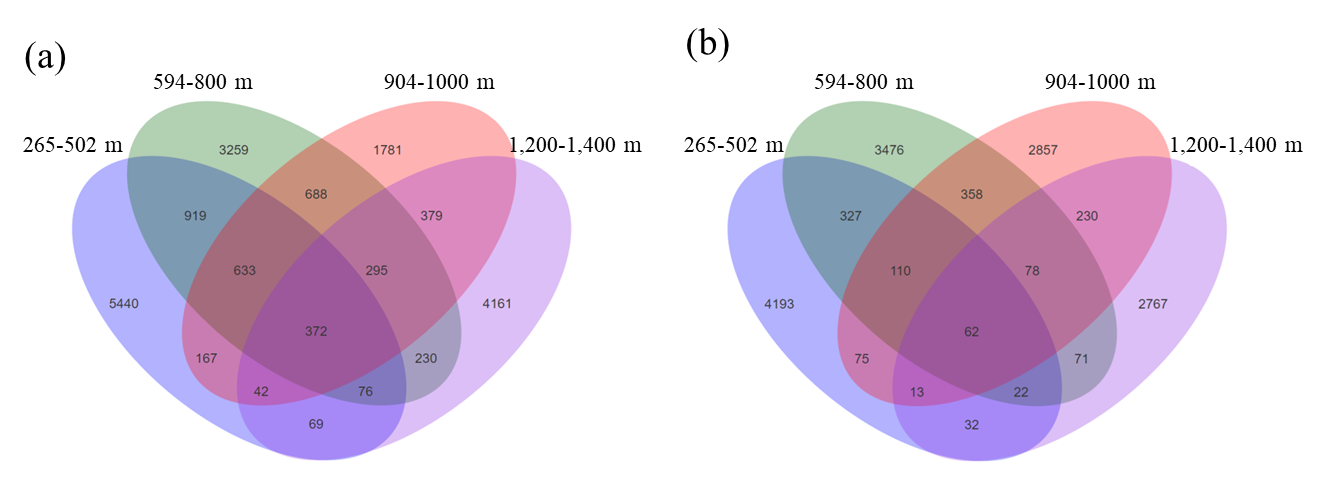


**Supplementary Figure 2.** Venn diagram shows the number of unique and shared ASVs among four elevational ranges. Cloud forests are located in the elevational rage s of 1,200-1,400 m.
